# Vasomotor responses to hypoxia and cold air

**DOI:** 10.1186/2046-7648-4-S1-A53

**Published:** 2015-09-14

**Authors:** Heather C Massey, James R House, Michael J Tipton

**Affiliations:** 1 Extreme Environments Laboratory, Department of Sport and Exercise Science, University of Portsmouth, Portsmouth, UK

## Introduction

At altitude, hypoxia coexists with other environmental stressors, in particular cold. Cold injury (CI) remains a frequent pathological consequence of exposure to altitude (>2800 m) [1]. A number of studies [2,3] have examined extremity vasomotor responses during local cold stress in controlled laboratory conditions at high altitude and have suggested that systemic arterial hypoxia exaggerates cold-induced cutaneous vasoconstriction and impairs any cold-induced vasodilatation (CIVD) response. Recently, Keramides *et al*. [4] reported that hypoxic exposure impairs the local rewarming response of the hands. In this way, hypoxia increases the risk of CI for a given temperature, but this hypothesis has not been tested in a dynamic air environment or during whole body exposure to the thermal stimulus, similar to that when at altitude. It is this dynamic response which determines the 'dose' of cold experienced by the extremities and thereby the risk of CI. It was hypothesized that vasoconstriction and vasodilatation would occur at warmer skin temperatures when breathing a hypoxic compared to normoxic gas mixture.

## Methods

Fourteen volunteers (males and females) gave their informed consent to participate in the ethically approved study during which they wore shorts and a t-shirt then inspired normoxic air (F_I_O_2_: 0.209 [N]), or a hypoxic gas mixture (F_I_O_2_: 0.113 [H]) in a balanced order. Throughout gradual cooling (-26 °C.hr^-1^) and rewarming (28.5 °C.hr^-1^) phases, skin temperatures (T_sk_) were measured continuously (at the chest, arm, thigh, exposed calf, right index finger and right great toe) with skin thermistors, and laser Doppler skin blood flow measured on the right great toe, little toe, thumb and little finger. Assessment of the onset and maximal vasoconstriction, and onset of vasodilatation were made by independent visual inspection by two researcers, using *a priori *definitions; mean skin temperatures (T_msk_) at these points were compared.

## Results

During the cooling phase, the onset of vasoconstriction of the thumb and little finger occurred at higher T_msk _H than N (thumb; H, 34.27 [0.78] °C, N, 33.83 [0.82] °C p=0.021; little finger; H 34.15 [1.06] °C, N, 33.37 [0.89] °C, p=0.009). Maximal vasoconstriction of the thumb and little finger occurred at higher T_msk _in hypoxia than normoxia (thumb; H, 32.00 [1.43] °C, N, 31.08 [1.31] °C p=0.025; little finger; H 32.39 [1.09] °C, N, 31.58 [1.25] °C, p=0.003). Greater T_sk _was observed at the onset of vasoconstriction in the little toe (H, 34.63 [0.85] °C, N, 33.87 [0.95] °C p=0.001), but was not seen at maximal vasoconstriction or at any point on the great toe. The onset of vasodilatation occurred at a warmer T_msk _in H compared to N in the thumb (H, 32.82 [1.88] °C, N, 32.01 [1.65], p=0.011) and little finger (H, 32.78 [1.95] °C, N, 32.05 [1.72] °C, p=0.006), but not in the great toe or little toe.

## Discussion

Acute exposure to normobaric hypoxia in combination with whole body cooling resulted in vasoconstriction at warmer Tsk compared to a normoxic condition in the hands but not the feet with the exception of the little toe. Similarly, the onset of vasodilatation occurred at higher T_sk _in hypoxia than normoxia in the hands only. Therefore, in general, the hypothesis can be accepted for the hands, but not the feet. The reason for the different responses of the hands and feet is not clear, but may relate to the greater sensitivity of the feet to cooling resulting in vasoconstriction at higher T_sk _in all conditions.

## Conclusion

Hypoxia and gradual cooling results in vasomotor responses which increase the 'dose of cold' experienced at the extremities, primarily the hands, and therefore increases the risk of CI.

## Acknowledgements

The authors would like to thank Gaizka Mejuto for his help on this project.
